# Robust Fourier transformed infrared spectroscopy coupled with multivariate methods for detection and quantification of urea adulteration in fresh milk samples

**DOI:** 10.1002/fsn3.987

**Published:** 2019-09-30

**Authors:** Fazal Mabood, Liaqat Ali, Ricard Boque, Ghulam Abbas, Farah Jabeen, Quazi Mohammad Imranul Haq, Javid Hussain, Ahmed Moahammed Hamaed, Zakira Naureen, Mahmood Al‐Nabhani, Mohammed Ziauddin Khan, Ajmal Khan, Ahmed Al‐Harrasi

**Affiliations:** ^1^ Department of Biological Sciences & Chemistry, College of Arts and Sciences University of Nizwa Nizwa Oman; ^2^ Department of Chemistry University of Sargodha Mianwali Pakistan; ^3^ Department of Analytical Chemistry and Organic Chemistry Universitat Rovira i Virgili Tarragona Spain; ^4^ Department of Chemistry University of Malakand Malakand Pakistan; ^5^ UoN Chair of Oman's Medicinal Plants and Marine Natural Products University of Nizwa Nizwa Oman

**Keywords:** milk adulteration, NIR spectroscopy, partial least‐squares discriminant analysis, partial least‐squares regressions, principal components analysis, urea

## Abstract

Urea is added as an adulterant to give milk whiteness and increase its consistency for improving the solid not fat percentage, but the excessive amount of urea in milk causes overburden and kidney damages. Here, an innovative sensitive methodology based on near‐infrared spectroscopy coupled with multivariate analysis has been proposed for the robust detection and quantification of urea adulteration in fresh milk samples. In this study, 162 fresh milk samples were used, those consisting 20 nonadulterated samples (without urea) and 142 with urea adulterant. Eight different percentage levels of urea adulterant, that is, 0.10%, 0.30%, 0.50%, 0.70%, 0.90%, 1.10%, 1.30%, and 1.70%, were prepared, each of them prepared in triplicates. A Frontier NIR spectrophotometer (BSEN60825‐1:2007) by Perkin Elmer was used for scanning the absorption of each sample in the wavenumber range of 10,000–4,000 cm^‐1^, using 0.2 mm path length CaF_2_ sealed cell at resolution of 2 cm^‐1^. Principal components analysis (PCA), partial least‐squares discriminant analysis (PLS‐DA), and partial least‐squares regressions (PLSR) methods were applied for the multivariate analysis of the NIR spectral data collected. PCA was used to reduce the dimensionality of the spectral data and to explore the similarities and differences among the fresh milk samples and the adulterated ones. PLS‐DA also showed the discrimination between the nonadulterated and adulterated milk samples. The *R*‐square and root mean square error (RMSE) values obtained for the PLS‐DA model were 0.9680 and 0.08%, respectively. Furthermore, PLSR model was also built using the training set of NIR spectral data to make a regression model. For this PLSR model, leave‐one‐out cross‐validation procedure was used as an internal cross‐validation criteria and the *R*‐square and the root mean square error (RMSE) values for the PLSR model were found as 0.9800 and 0.56%, respectively. The PLSR model was then externally validated using a test set. The root means square error of prediction (RMSEP) obtained was 0.48%. The present proposed study was intended to contribute toward the development of a robust, sensitive, and reproducible method to detect and determine the urea adulterant concentration in fresh milk samples.

## Graphical abstract

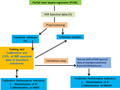

## 1 INTRODUCTION

The analytical methods resulting from the use of the NIR spectroscopic region reflect some significant characteristics such as fast, nondestructive, noninvasive, with high penetration of the probing radiation beam, suitable for in‐line use, nearly universal application (any molecule containing C‐H, NH, S‐H, or O‐H bonds), and with minimum sample preparation demands. The combination of these characteristics with instrumental control and data treatment has made it possible to coin the term Near‐Infrared Technology (Celio, 2003).

Milk and other dairy products are consumed all around the globe and are highly nutritious (De Toledo, Toci, Pezza, & Pezza, 2017). Milk has been considered the “complete food” as it contains enough essential nutrients required by infants, children, and adults (Park & Haenlein, 2013). It is a key source for proteins, fats, carbohydrates, vitamins, and minerals (De La Fuente & Juarez, 2005; Pehrsson, Haytowitz, Holden, Perry, & Beckler, 2000). Calcium and phosphorus are the main mineral nutrients of milk that are required for growth of newly borne babies and essential for stronger bones in human (Dobrzański, Kołacz, Górecka, Chojnacka, & Bartkowiak, 2005; Mabood et al., 2017; Malbe, Otstavel, Kodis, & Viitak, 2010). The good effects of milk proteins on human health include anti‐microbial, immunomodulatory, anti‐thrombotic, antihypertensive activities, and antioxidative (Antanasova & Ivanova, 2010).

Unfortunately, milk is one of the most vulnerable targets for economically motivated adulteration (Moore, Spink, & Lipp, 2012) and these adulterants cause serious illnesses to the consumers which may lead to death in some cases. The milk adulterants include mainly the vegetable proteins, whey, watering, and milk from different species (Singh & Gandhi, 2015). The major hazardous adulterants of milk include urea, formalin, ammonium sulfate, boric acid, detergents, caustic soda, salicylic acid, hydrogen peroxide, benzoic acid, melamine, and sugars. Urea adulteration up to 500 mg/L can result into cancer and failure of kidneys (De et al. 2011; De Toledo et al., 2017). The allowed limit for the presence of urea in fresh milk by Some researchers has recommended a range of 10–14 milligrams per deciliter (mg/dl) while others have recommend at range of 8–12 mg/dl (Penn State Extension report). Cow milk containing urea as contaminant has been reported to cause ulcer, acidity, kidney stones, and indigestion (Ezhilan et al., 2017), as urea adulterated milk is considered to overburden the kidneys (Kandpal, Srivastava, & Negi, 2012). The milk adulterated with excessive starch can accumulate undigested starch in colon, which can cause diarrhea and in some cases can also lead to fatality in diabetic patients (Singuluri & Sukumaran, 2014).

Adulteration or adding illegal additives to food products is becoming a global issue for the consumers. Due to lack of adequate monitoring policies, the underdeveloped and the developing countries are prone to higher risk of human health (Azad & Ahmed, 2016). Various optical detection techniques (Castillo‐Ortega et al., 2002; Lakard, Herlem, Lakard, Antoniou, & Fahys, 2004) have been explored for the qualitative and quantitative detection of adulteration in milk. Examples include fluorescence spectroscopy (Xiang, Zeng, Zhai, Li, & He, 2011), Raman spectroscopy (Khan, Krishna, Majumder, & Gupta, 2015; Okazaki, Hiramatsu, Gonmori, Suzuki, & Tu, 2009), nuclear magnetic resonance (NMR) spectroscopy (Hilding‐Ohlsson, Fauerbach, Sacco, Bonetto, & Cortón, 2012), diffuse reflectance spectroscopy (De Toledo et al., 2017), and MID‐IR spectroscopy (Kishor & Thakur, 2015). Colorimetric sensor array (Yang, Huo, Jiang, Hou, & Zhang, 2013), voltamperometric method (Hilding‐Ohlsson et al., 2012), and enzyme‐linked immunosorbent assay (ELISA) (Garber, 2008) have also been used as the sensing techniques for milk adulterant detection (Kamal & Karoui, 2015; Tittlemier, 2010).

Nowadays, milk adulteration is being carried out more sophisticatedly (Azad & Ahmed, 2016), whereas the standard methods for food protein analysis rely mainly on the measurement of nitrogen content by using classical detection techniques (Garcia et al., 2012). Therefore, it has become difficult to differentiate the adulterant nitrogen from the milk protein and the nitrogen‐rich chemicals commonly used as the adulterants (Qin et al., 2017). Hence, there is a direct need for cutting edge research through dissemination and implementation of more advanced techniques to detect these adulterants. In a previous study, we reported (Mabood et al., 2017) a NIRS method coupled with chemometrics to authenticate the level of adulteration of goat milk in camel milk. The present study was intended to contribute toward the development of a robust, highly sensitive, and reproducible Fourier transformed infrared spectroscopy (FT‐NIRS) with the help of application of chemometric methods method to determine the urea adulterant concentration in cow milk. The fresh cow milk samples were intentionally adulterated with various concentrations of commercial urea and then submitted to NIR spectral measurements. Multivariate analysis was finally applied to authenticate and quantify the levels of adulteration.

## 2 MATERIALS AND METHODS

### 2.1 Preparation of the urea adulterated fresh milk samples

In this study, 162 fresh milk samples were used, those consisting of 20 nonadulterated samples (without urea) and 142 with urea adulterant. Eight different percentage levels of urea adulterant, that is, 0.1%, 0.3%, 0.5%, 0.7%, 0.9%, 1.1%, 1.3%, and 1.7%, each of them prepared in triplicates, were used. The measured NIR spectral data were split into two sets. A training set including 70% of the data was used for building the PLSR model, while the second set was the test set including 30% of the spectra and used for external validation of the PLSR model.

### 2.2 Fourier transform near‐infrared spectroscopic analysis

A Frontier NIR spectrophotometer (BSEN60825‐1:2007) by Perkin Elmer was used for measuring the absorption of each milk sample in the wavenumber range of 10,000–4,000 cm^‐1^, using 0.2 mm path length CaF_2_ sealed cell at a resolution of 2 cm^‐1^.

### 2.3 Multivariate analysis

Principal components analysis (PCA), partial least‐squares discriminant analysis (PLS‐DA), and partial least‐squares regressions (PLSR) methods were applied for the multivariate analysis of the measured NIR spectral data using the Unscrambler version 9.00 and Microsoft Excel 2010 softwares. PCA was used to reduce the dimensionality of the spectral data and to explore the similarities and differences among the fresh milk samples from the ones adulterated with urea. PLS‐DA was used to discriminate between adulterated and nonadulterated milk samples. Furthermore, the PLSR models were also built to quantify the levels of urea in the fresh milk samples. The PLSR model was externally validated using the test set of samples.

## 3 RESULTS AND DISCUSSION

### 3.1 NIR spectra

**Figure 1:**
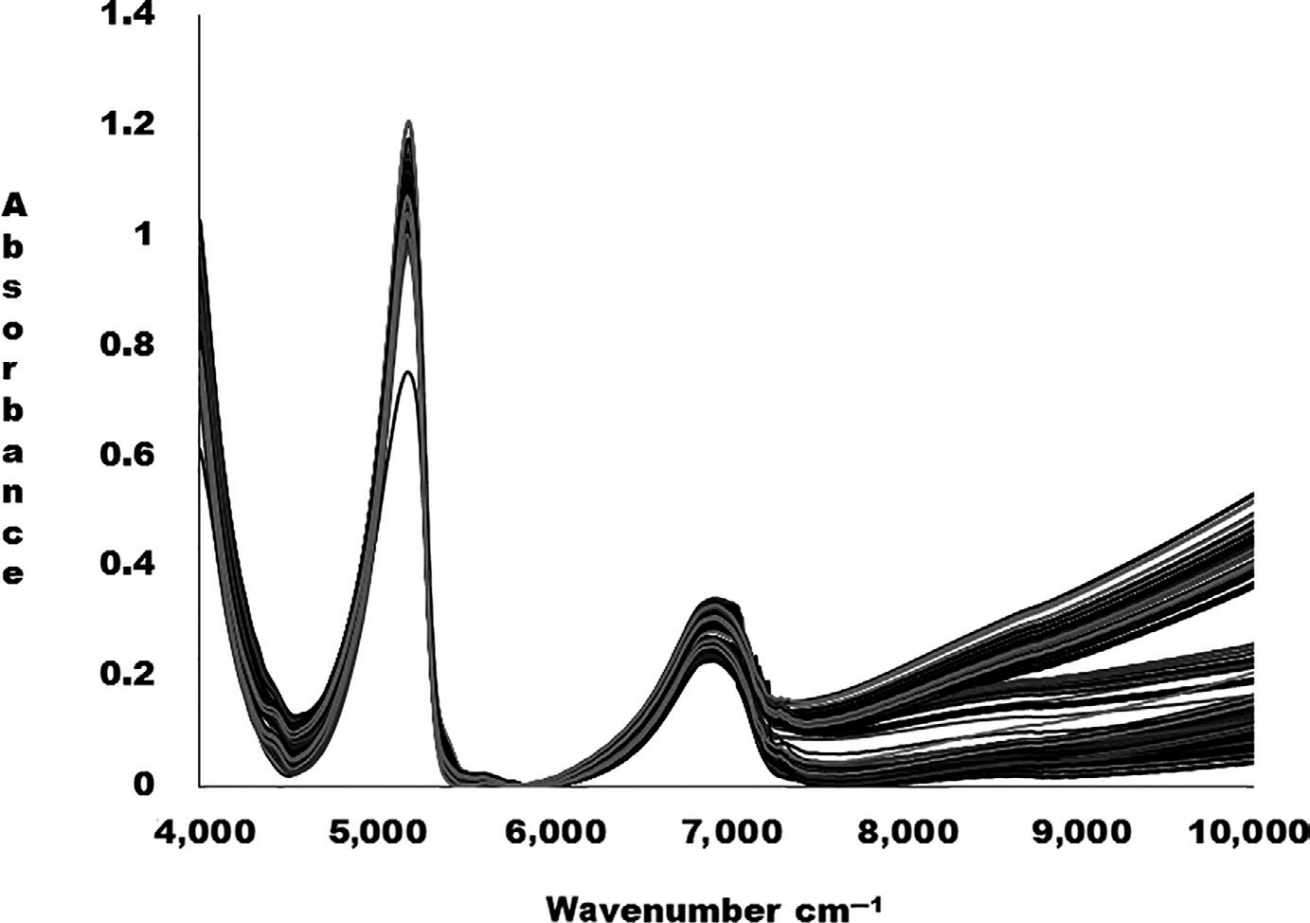
Raw (not preprocessed) NIR spectra for all the adulterated and unadulterated fresh milk samples

The actual NIR spectral data obtained by running all the adulterated and nonadulterated fresh milk samples through FT‐NIR spectrophotometer are shown in Figure 1.

Prior to the application of various chemometric methods on the near‐infrared spectral data, spectral transformations such as baseline correction, 1st derivative with Savitzky–Golay smoothing, and standard normal variate (SNV) were also applied. The preprocessing on the NIR spectra was applied to remove the noise and to minimize the effect of scattering due to the presence of the suspended particles in fresh milk samples (see Table 1). The selection of the optimal spectral transformations was based on the values of the *R* ^2^, RMSE, and RMSEP of the PLSR models, the best preprocessing spectral treatment being the one with minimum values of RMSE, RMSEP and number factors, and maximum value of *R* ^2^.

**Table 1:** Selection of preprocessing spectral treatment

Type of spectra	Preprocessing	PLS		PLS		No. of factors
		RMSE	*R* ^2^	RMSEP	*R* ^2^	
Full Spectra (4,000–10,000 cm^−1^)	Without spectral transformations	0.680	0.988	0.552	0.989	4
Spectra from (4,000–7,300 cm^−1^)	Without spectral transformations	0.668	0.991	0.568	0.989	4
Full Spectra (4,000–10,000 cm^−1^)	Baseline	0.627	0.986	0.631	0.986	5
Spectra (4,000–7,300 cm^−1^)	Baseline	0.592	0.988	0.623	0.987	5
Full Spectra (4,000–10,000 cm^−1^)	SNV	0.684	0.984	0.606	0.987	5
Spectra (4,000–7,350 cm^−1^)	SNV	0.635	0.986	0.553	0.989	5
Full Spectra (4,000–10,000 cm^−1^)	1st derv. spectral transformations	0.649	0.985	0.760	0.980	3
Spectra (4,000–6,400 cm^−1^)	1st derv. spectral transformations	0.563	0.987	0.483	0.983	3

PLS‐DA: partial least‐squares discriminant analysis; RMSEP: root means square error of prediction; SNV: standard normal variate.

As it can be seen from Table 1, the 1st derivative function with S‐Golay including 11 smoothing points in the wavenumber range of 4,000–6,400 cm^‐1^ is the optimal spectral transformation for building the PLSR model. The preprocessing was optimized using the criteria based on *R* ^2^, RMSE, RMSEP, and less number of PC or factors. The optimum PLSR model is the one for that the value of coefficient of determination *R* ^2^ is maximum close to 1, while the error values like RMSE and RMSEP are minimum as well as the number of PC or factors are also less.

**Figure 2:**
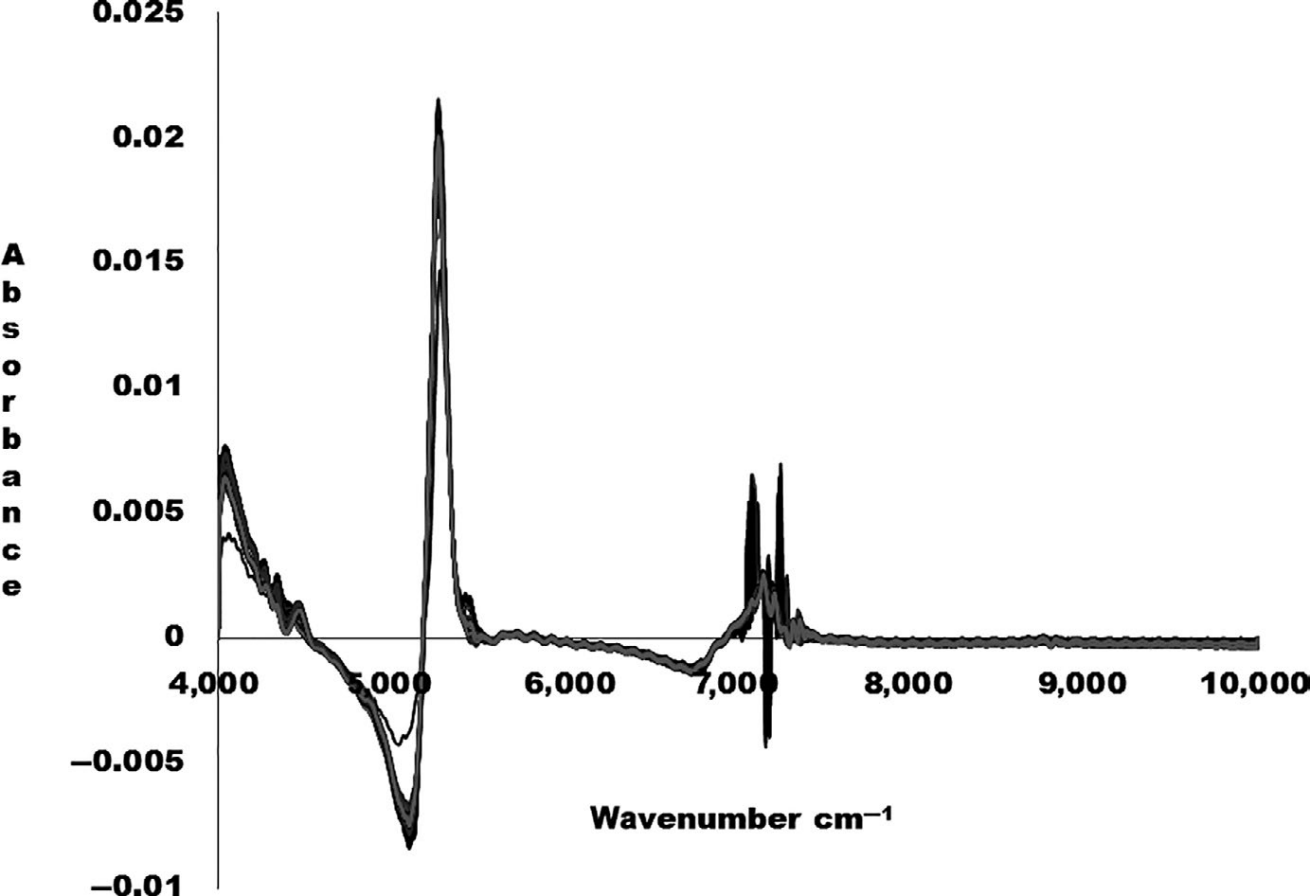
The 1st derivative transformed NIR spectra for the fresh milk samples.

The 1st derivative–transformed NIR spectra for milk samples are shown in Figure 2.

From the wavenumber 4,000–6,000 cm^‐1^, there are prominent absorption peaks as shown in Figure 2. The NIR spectra for fresh milk samples are difficult to interpret due to the presence of several chemical compounds that may cause band overlap. The dominating broad band region between 6,000 and 7,500 cm^‐1^ represents symmetric and anti‐symmetric stretching modes of water. The strong water band obscured the protein band in this region. The weak band at 5,628 cm^‐1^ could be due to C‐H stretching mode of CH_2_ and C‐H groups of fat. The absorption band at 5,255 cm^‐1^ is related to O‐H stretching of H‐OH deformation mode of polysaccharides. The region between 4,000 and 4,600 cm^‐1^ can be associated to the combination of the CH_2_ vibration of the protein side chain (Šašić & Ozaki, 2000; Sivakesava & Irudayaraj, 2002).

**Figure 3:**
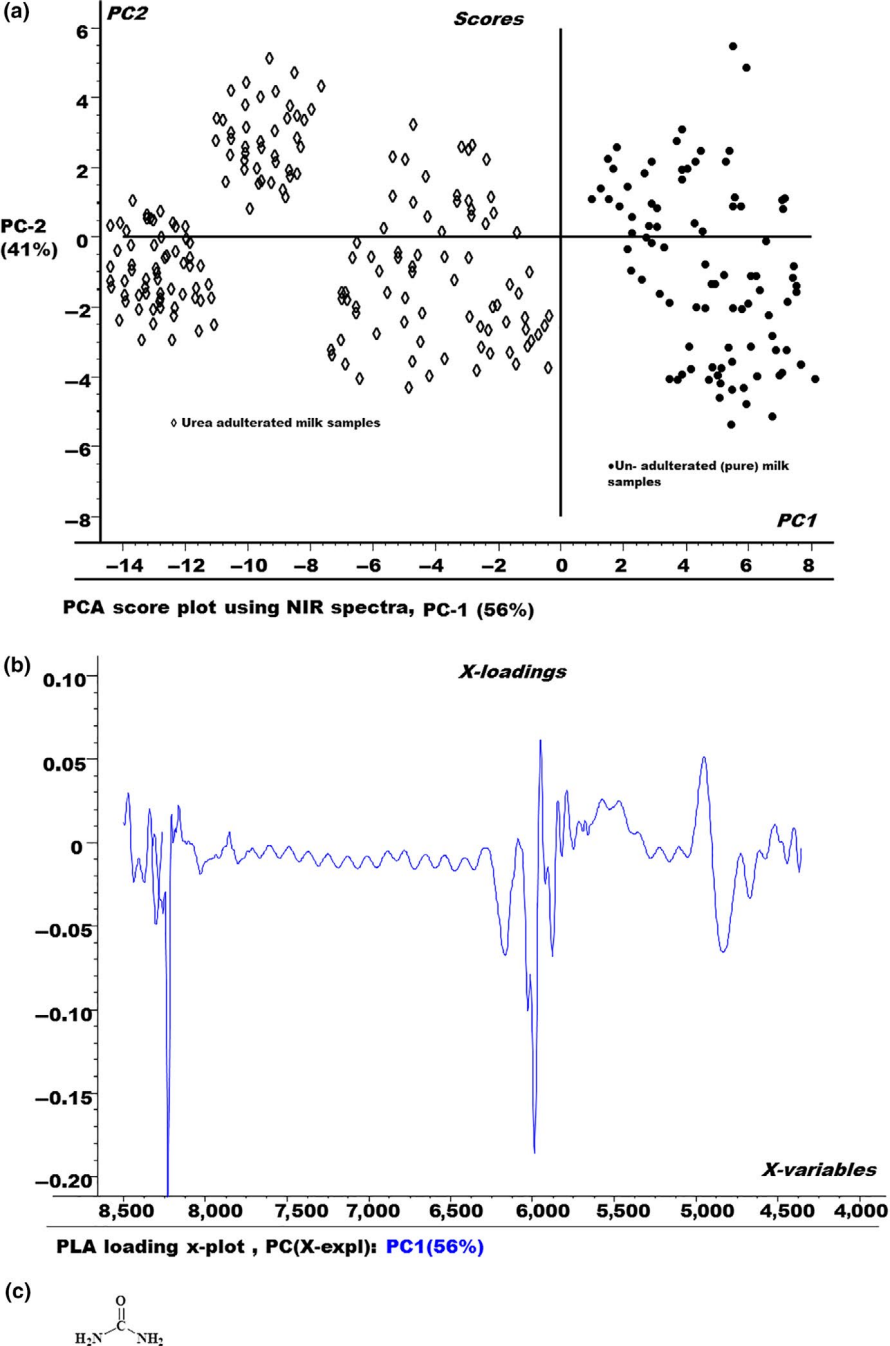
(a) The principal components analysis (PCA) score plot for urea adulterated and nonadulterated fresh milk samples. (b) PCA loading plot for urea adulterated and nonadulterated fresh milk samples. (c) Chemical structure of urea

Chemometrics in this scenario can provide the solution through empirical models to overcome these problems, by relating the multiple spectral intensities from the numerous regression samples to known parameter values of these samples. A PCA model was applied on the NIR spectra to reduce the dimensionality and to explore the similarities and differences among the samples, both adulterated and nonadulterated, as shown in Figure 3.

The score plot of PCA in Figure 3a shows a complete segregation of urea adulterated milk samples from the pure milk samples. All the urea adulterated milk samples are appearing on the left region of the PCA score plot as compared to nonadulterated milk samples. This indicates that urea adulterated milk samples have a different absorption pattern as compared to pure milk samples, due to the presence of urea as adulterant.

Figure 3b shows the PCA X‐loading plot for PC1. It describes how much of the difference in a spectral variable is explained by the PC. In this case, PC contain 56% of the total spectral variation X. It also tells about the spectral regions those contribute more to the PCA model.

The chemical structure of the urea molecule is shown in Figure 3c.

The absorbance spectrum of the urea exhibited two broad absorption bands at 4,650 and 4,550 cm^‐1^ is associated with symmetric (3,350 cm^‐1^) and asymmetric (3,450 cm^‐1^) stretching bands of N‐H coupled with the bending vibration (1,640–1,600 cm^‐1^) of N‐H. In the aqueous environment, a prominent shift is observed in vibrational frequencies due to hydrogen bonding. For the combination bands of urea, magnitude of red shift is 400 cm^‐1^, which is evident and consistent with earlier reports for amides in aqueous solution (Eddy & Arnold, 2001; Edward & Mahpour, 1973; Silverstein, Bassler, & Morrill, 1991).

**Figure 4:**
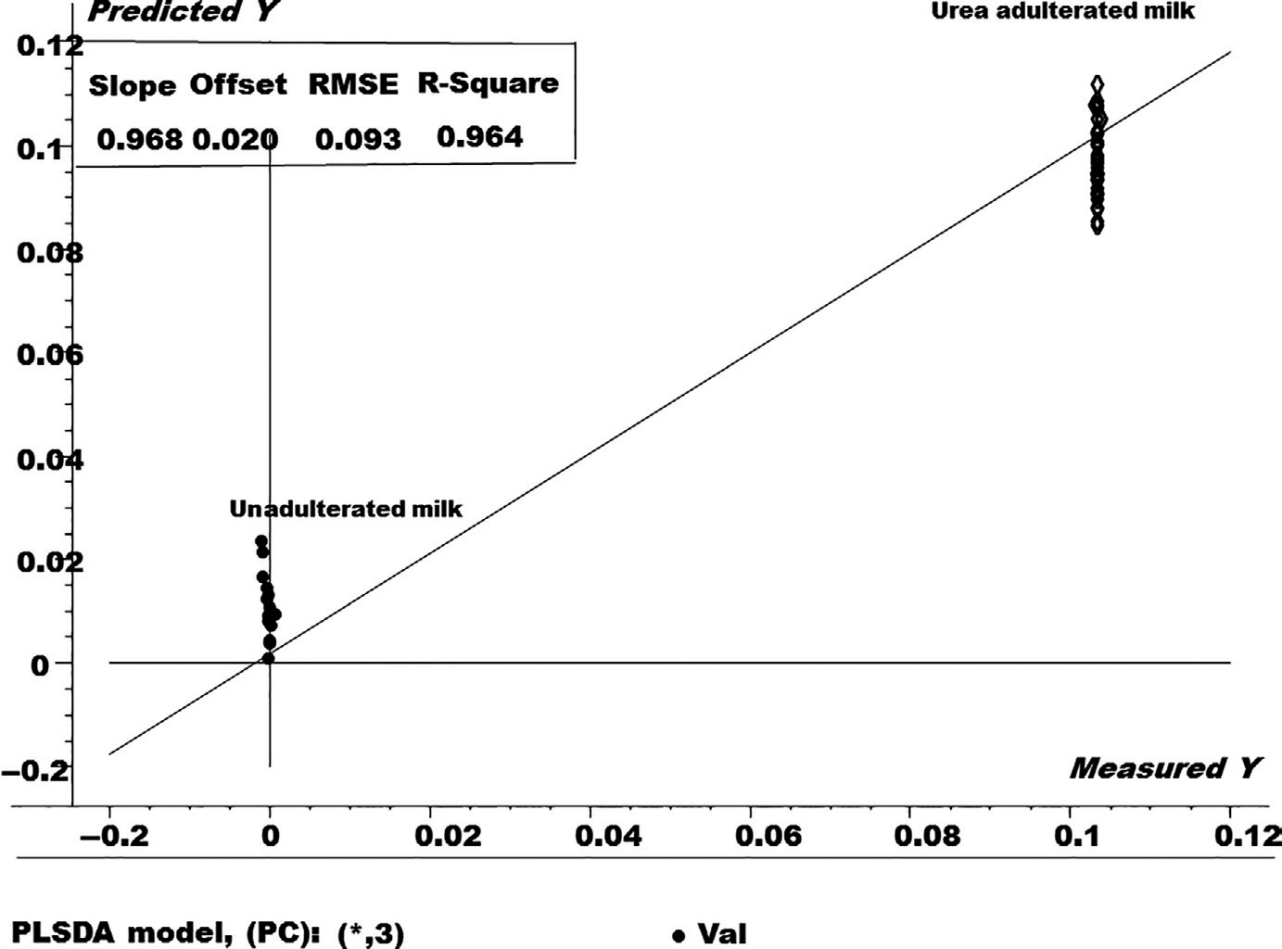
The partial least‐squares discriminant analysis model for urea adulterated and nonadulterated fresh milk samples

Similarly, the PLS‐DA model was also showed the discrimination between the milk samples, as shown in Figure 4.

The PLS‐DA model in Figure 4 shows that the 0% concentration of the urea adulterant (pure fresh milk) are completely discriminated from those adulterated with 0.1% urea adulterant. Therefore, it can be used as a tool for the robust detection of urea adulterant in the fresh milk samples. The *R*‐square value obtained for the PLS‐DA model was 0.9680 with 0.08% value of the root mean square error (RMSE).

**Figure 5:**
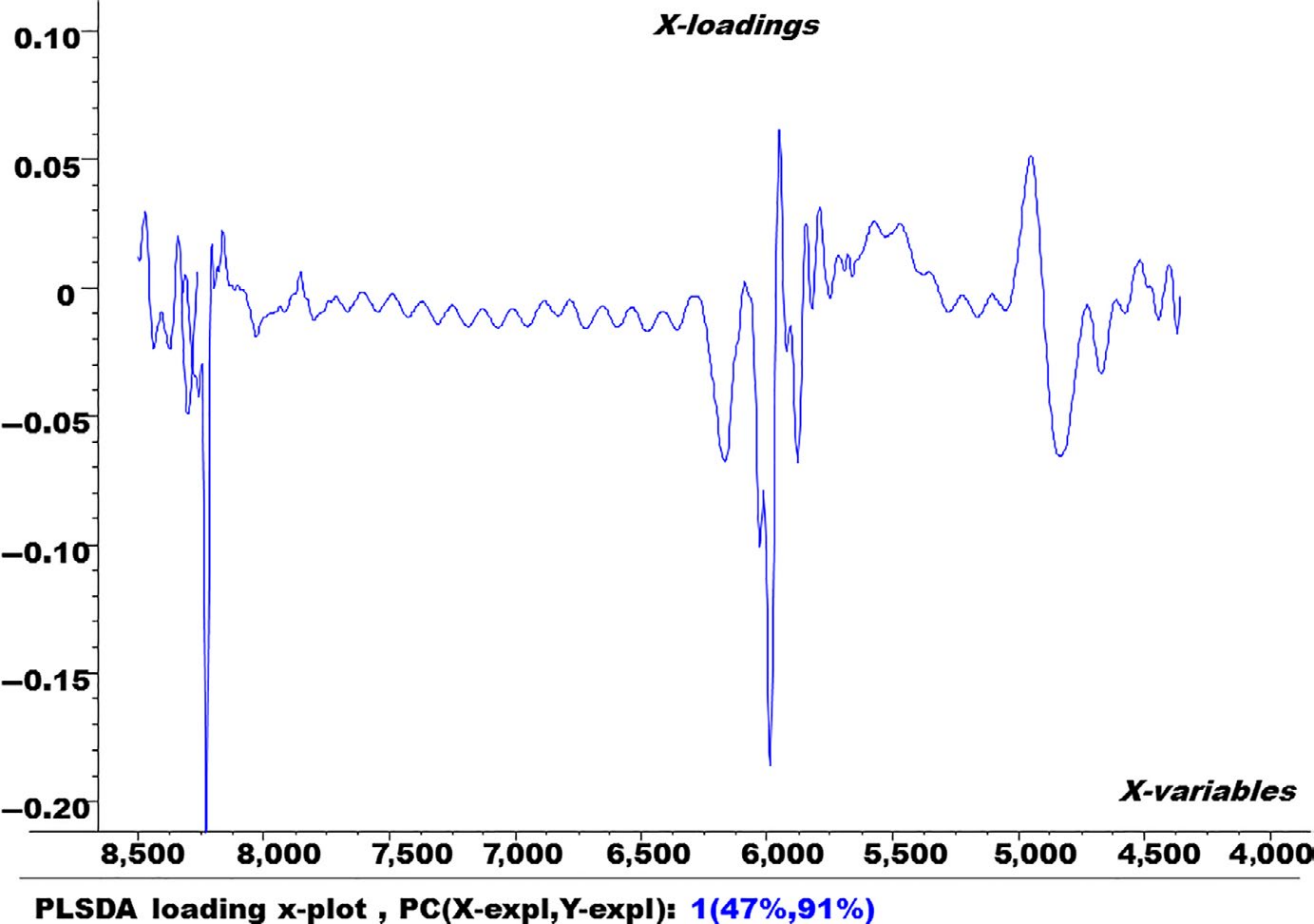
Factor loading plot of the partial least‐squares discriminant analysis model for factor 1

In order to see the variation in the spectral data during building the PLS‐DA model, the x‐factor loading plot was also built and shown in Figure 5.

Figure 5 illustrates that 47% of the spectral variation was used in building the PLS‐DA model by factor 1. It also indicates the wave numbers that contributed the most to building this model.

### 3.2 PLS regression results

**Figure 6:**
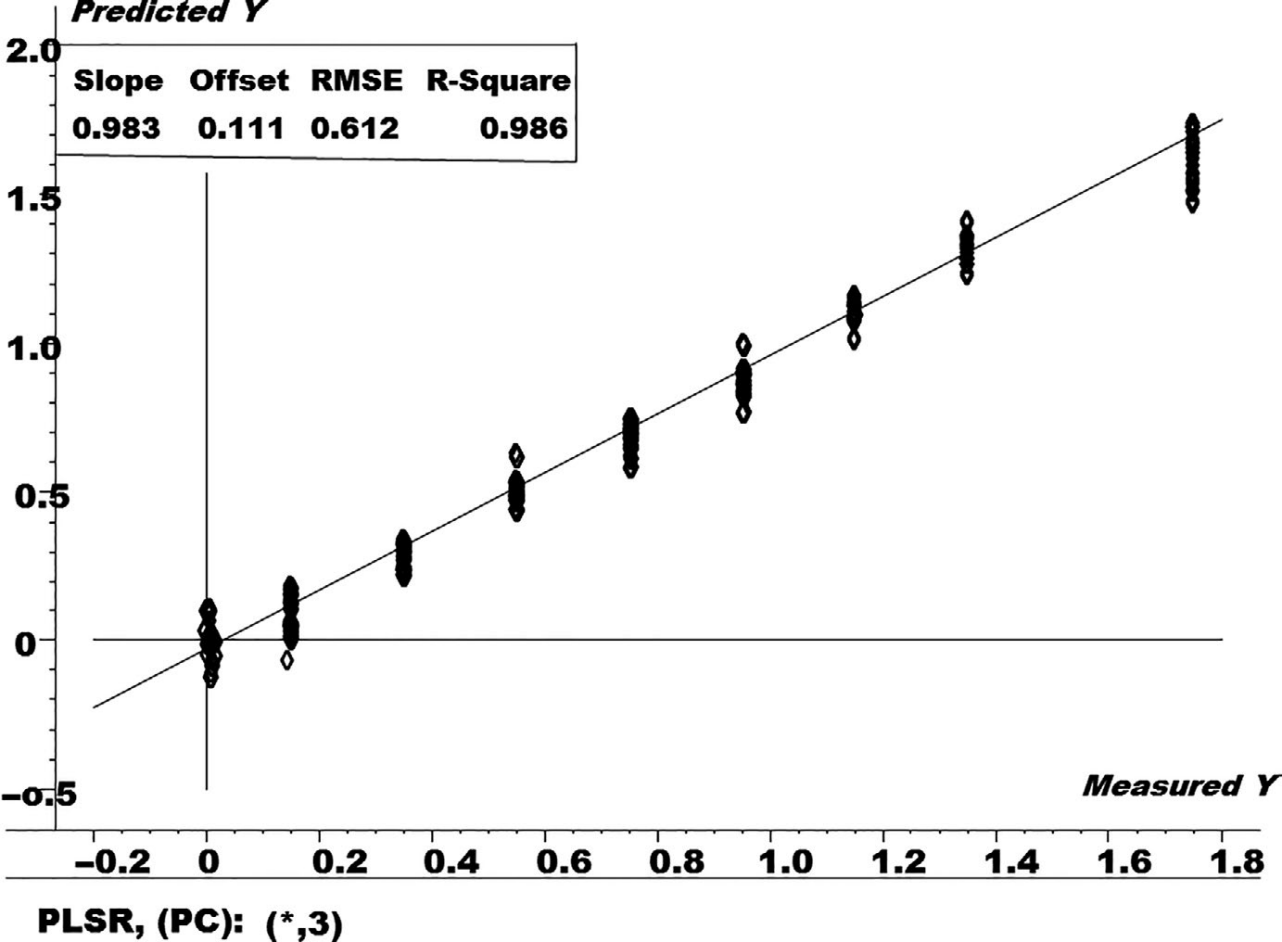
Partial least‐squares regressions (PLSR) regression plot for pure and urea adulterated fresh milk samples

Furthermore, a PLSR model was also built on the NIR spectral data in order to quantify the levels of the urea adulterant in the fresh milk samples, as shown in Figure 6. The PLSR model was built by using 70% of the NIR spectral data, that is, the training set.

PLS regression model makes a set of orthogonal components that maximizes the level of correlations in between both the NIR spectral data, that is, **X,** and the concentration, that is, **Y,** and provide a predictive equation for **Y** in terms of the **X**'s for future unknown samples.

**Figure 7:**
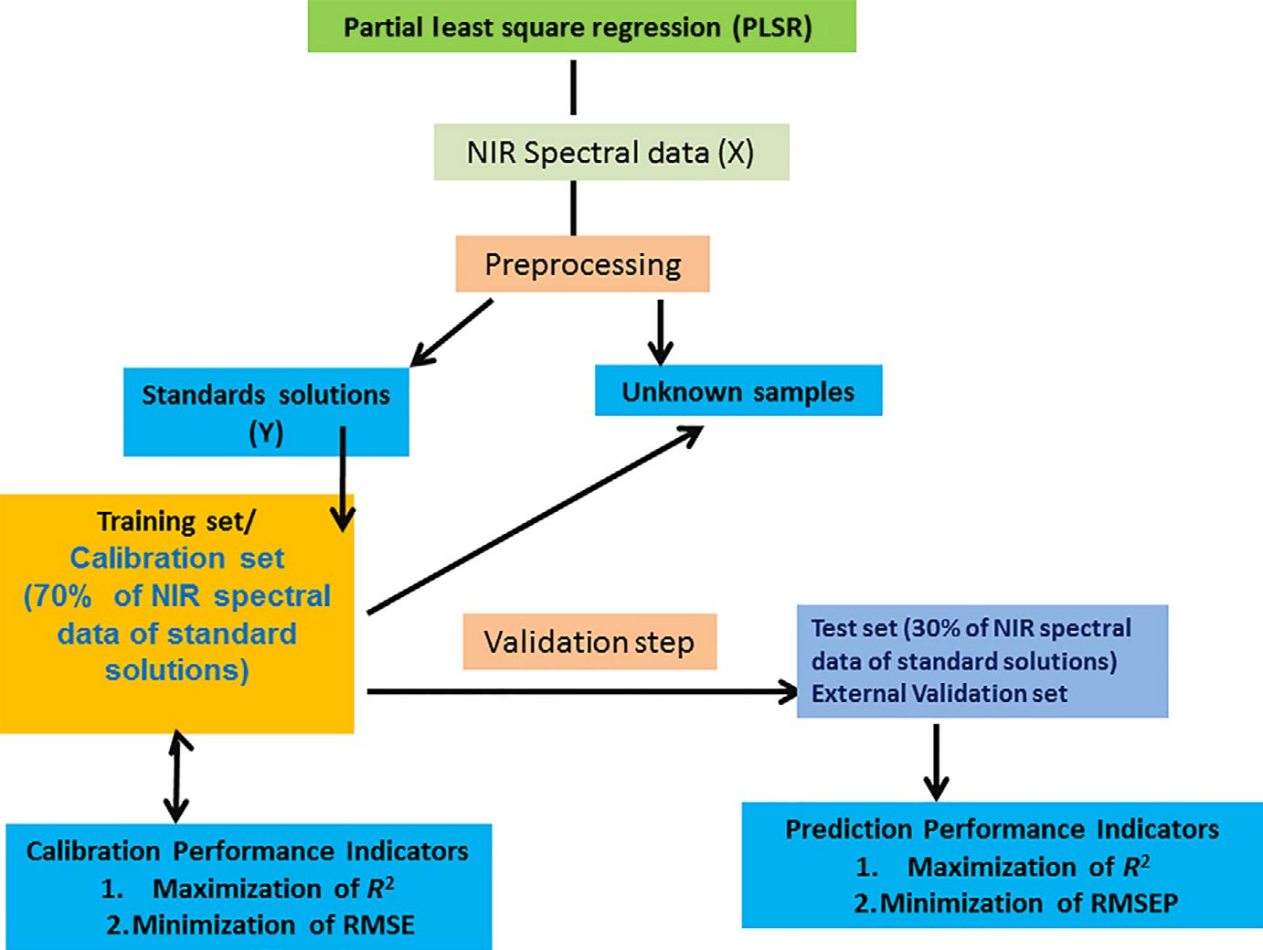
Generalized flow chart of partial least‐squares regression analysis

Figure 7 shows the generalized procedure as well as validation methods of the multivariate PLS regression analysis applied on the obtained NIR spectral data. It shows that the NIR spectral data of all the adulterated and nonadulterated fresh milk samples were first transformed with the application of 1st derivative spectral pretreatment. After that, the spectral data of the urea adulterated milk samples (reference samples/standards) were split into two set: training set and test set. The training set was used for building the PLS regression model while the test set was used as a test set to check the performance of PLS regression model for validation. After external validation, it was applied to unknown fresh milk samples. Here, two types of validation, that is, internal validation using leave on out on training set and external validation using the test set, were performed.

The *R*‐square and root mean square error (RMSE) values for the PLSR model in Figure 6 were found to be 0.986 and 0.612%, respectively. The RMSE is a statistical measure used to check the prediction ability of the PLSR model, using “pseudo” external samples and using the leave‐one‐out procedure. The best PLSR model is the one which has the smaller value of RMSE along with high value of correlationship. It is calculated as in Equation (1):

(1) $$\text{RMSE =}\;\sqrt{\frac{\sum_{i=1}^{n}{\left(y_{i}-{\hat{y}}_{i}\right)}^{2}}{n}}$$

where *y_i_* is the measured value (actual % of adulteration), ${\hat{y}}_{i}$ is the % of adulteration predicted by the model, and *n* is the number of segments left‐out in the cross‐validation procedure, which is equal to the number of samples of the training set. Smaller the value of RMSE is a better indicator for the prediction ability of the PLSR model.

**Figure 8:**
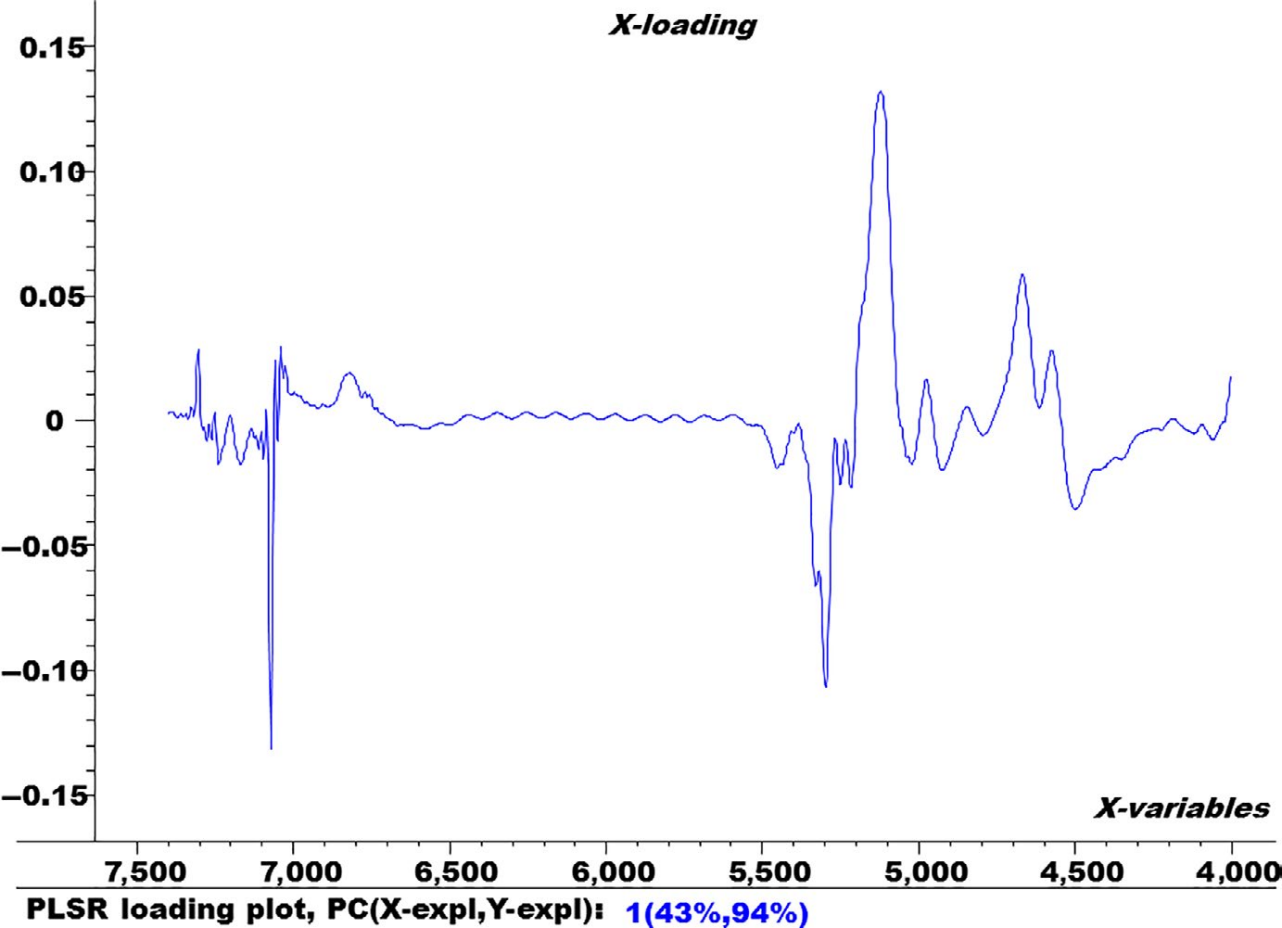
Factor loading plot for factor 1

In order to show the variation in the spectral data during building the PLSR model, the factor loading plot is shown in Figure 8.

Figure 8 shows the factor loading plot for factor 1 of the optimum PLSR model. It shows that factor 1 loading plot contributes 43% and 94% to the modeling of X (spectra) and Y (% urea), respectively. The factor loading plot shows what spectral variables (wavenumbers) contribute more to building the PLS regression model. This loading plot exhibited that the two broad absorption bands at 4,650 cm^‐1^ and 4,550 cm^‐1^ are associated with symmetric and asymmetric stretching bands of N‐H of urea.

**Figure 9:**
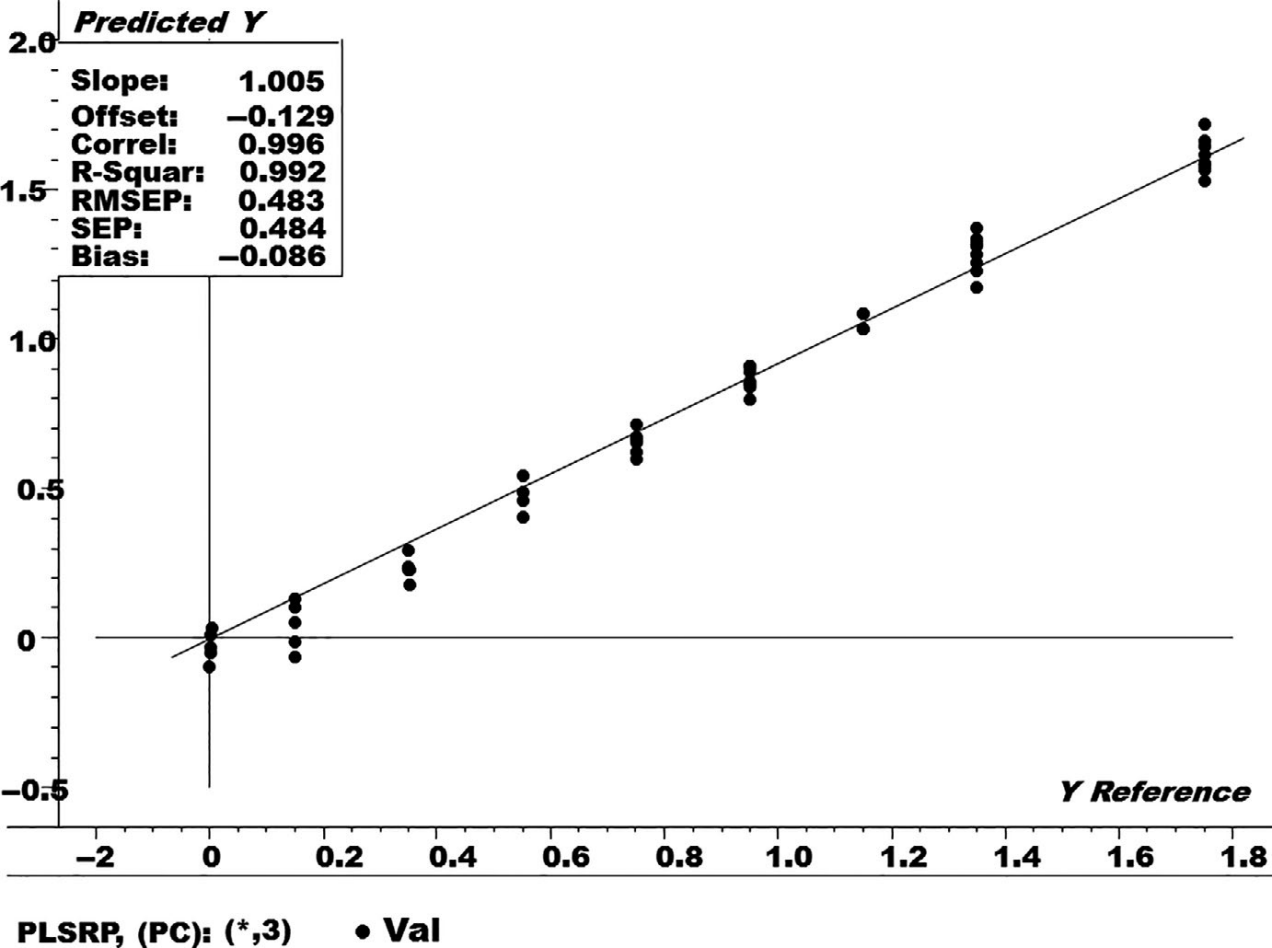
Partial least‐squares prediction plot for the test set of fresh milk samples

Once the PLS regression model was established, it was then assessed using the external test set including 30% the NIR spectral data, as shown in Figure 9.

Figure 9 shows that the PLS regression model displayed a very good prediction ability, with prediction error, that is, (RMSEP = 0.483%) with a high correlation coefficient (*R* = 0.99). The RMSEP is a statistical measure used to assess the prediction ability of the PLS model with totally new samples (not used during the calibration process), and it is calculated using Equation (2):

(2) $$\text{RMSEP =}\;\sqrt{\frac{\sum_{i=1}^{n_{t}}{\left(y_{t,i}-{\hat{y}}_{t,i}\right)}^{2}}{n_{t}}}$$

where *y_t,i_* is the measured value (actual % of adulteration), ${\hat{y}}_{t,i}$ is the % of adulteration predicted by the model, and *n_t_* is the number of samples in the test set. RMSEP expresses the average error to be expected in future predictions when the calibration model is applied to unknown samples.

Based on the minimum value of RMSEP (model with three factors), the PLS regression model can be applied to unknown fresh milk samples for detection and quantification of urea adulteration in any fresh milk sample.

## 4 CONCLUSION

The results gleaned from this study revealed that NIR spectroscopy coupled with multivariate methods can be deployed as a robust, sensitive, and nondestructive technique for detecting and quantifying the presence of urea adulteration in various fresh milk samples. The current study revealed that PLS‐DA model can be used to discriminate between the milk samples those were adulterated with urea from the fresh milk samples (unadulterated). Furthermore, the PLSR models may be used to quantify the level of the urea adulterant in milk samples (https://extension.psu.edu/interpretation-of-milk-urea-nitrogen-mun-values).

## CONFLICT OF INTEREST

The authors declare that they do not have any conflict of interest.

Ethical Review: This study does not involve any human or animal testing.

Informed Consent: Written informed consent was obtained from all study participants.
